# CD8 Encephalitis: A Diagnostic Dilemma

**DOI:** 10.3390/diagnostics12112687

**Published:** 2022-11-04

**Authors:** Rohan Sharma, Thomas Spradley, Morgan Campbell, Shubham Biyani, Pulkit Singhal, Hisham Elkhider, Krishna Nalleballe, Murat Gokden, Manoj Kumar, Nidhi Kapoor

**Affiliations:** 1 Department of Neurocritical Care, Mayo Clinic in Florida, Jacksonville, FL 32224, USA; 2 Department of Medicine, University of Arkansas for Medical Sciences, Little Rock, AR 72205, USA; 3 College of Medicine, University of Arkansas for Medical Sciences, Little Rock, AR 72205, USA; 4 Department of Neurology, University of Arkansas for Medical Sciences, Little Rock, AR 72205, USA; 5 College of Osteopathic Medicine, California Health Sciences University, Clovis, CA 93612, USA; 6 Department of Pathology, University of Arkansas for Medical Sciences, Little Rock, AR 72205, USA; 7 Department of Radiology, University of Arkansas for Medical Sciences, Little Rock, AR 72205, USA; 8 Department of Neurology, Baptist Medical Center, Jacksonville, FL 32207, USA

**Keywords:** CD8 encephalitis, HIV, HSV, encephalitis

## Abstract

CD8+ encephalitis is a subacute encephalopathy associated with HIV infection. Pathophysiology is thought to be auto-reactive CD8+ cells attacking on HIV infected CD4+ cells and ‘viral escape’ phenomena (replication of CD8+ cells in CSF). We present a case of a 45-year-old man with well controlled HIV who developed CD8 encephalitis following Herpes simplex encephalitis. He had persistent encephalopathy for several weeks with status epilepticus and agitated delirium, and diagnosis remained elusive until a brain biopsy confirmed the diagnosis.

## 1. Case

### 1.1. Admission 1

A 45-year-old man with well-controlled HIV (CD4 count one year prior of 575/μL (40%) and viral load <20 copies/mL) on bictegravir, emtricitabine, and tenofovir alafenamide; hepatitis C (previously treated); liver cirrhosis; prior polysubstance abuse (including methamphetamine, cannabis, cocaine and ETOH); presented with new-onset seizure and confusion. Given the patient’s confusion, history was obtained from the patient’s husband. Patient reportedly had nausea and vomiting after an episode of binge drinking for three days followed by new-onset seizures. He had daily seizures for three days which caused him to seek medical attention. There was no history of similar events. Patient reportedly had a tick bite a few weeks prior to presentation. He did not report any fever, chills, rash, headaches, vision changes, cough, diarrhea, or abdominal pain.

On presentation he was found to be febrile at 102.9 F (39.3 C) and tachycardic at 115/min, with a normal blood pressure of 119/52 mmHg and respiratory rate of 18/min. His cardiorespiratory and abdominal exams were unremarkable. He was drowsy, arousable with minimal stimuli, and confused, but he followed simple commands without any cranial nerve or sensory-motor deficits or neck rigidity. His labs were significant for a neutrophil-predominant leukocytosis of 12,000/mm^3^, hyponatremia of 133 mg/dL, and an elevated creatinine of 1.25 mg/dL. His chest radiograph was unremarkable, and a CT head without contrast was also unremarkable. His CSF studies showed a lymphocyte-predominant pleocytosis: 162 (97% monocytes), with a protein of 45 mg/dL, and a glucose of 77 mg/dL. He was given supplemental oxygen by nasal cannula; IV acyclovir, ceftriaxone, and vancomycin for presumed meningitis/encephalitis; doxycycline for possible tick-bone illness; and levetiracetam for seizures. He was also given thiamine for possible Wernicke’s encephalitis secondary to alcohol use. Electroencephalogram (EEG) showed left focal temporal slowing.

On day two of admission, the patient became tremulous, anxious, and hypoxic with an arterial blood gas showing acidosis, hypoxia, and hypercarbia: pH 7.27, pCO_2_ 46 mmHg, pO_2_ 78 mmHg. He had an elevated anion gap of 25. A chest radiograph was normal. He was transferred to the intensive care unit for hypoxic respiratory failure and started on as-needed lorazepam and dexmedetomidine for agitation and alcohol withdrawal. CT chest with contrast, pulmonary embolus (PE) protocol, did not show any PE but showed patchy airspace opacities in bilateral lungs, which was most extensive in the lung bases. CT abdomen with contrast revealed cholelithiasis with a thickened gallbladder wall, diffuse circumferential thickening of the esophagus, cirrhotic liver morphology with multiple splenic and gastric varices, splenomegaly, and multiple small bilateral renal calculi.

**Figure 1:**
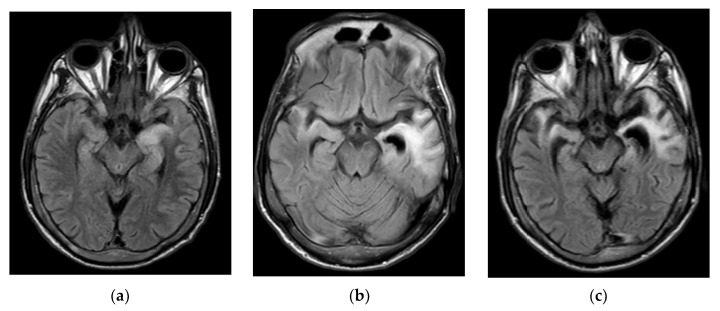

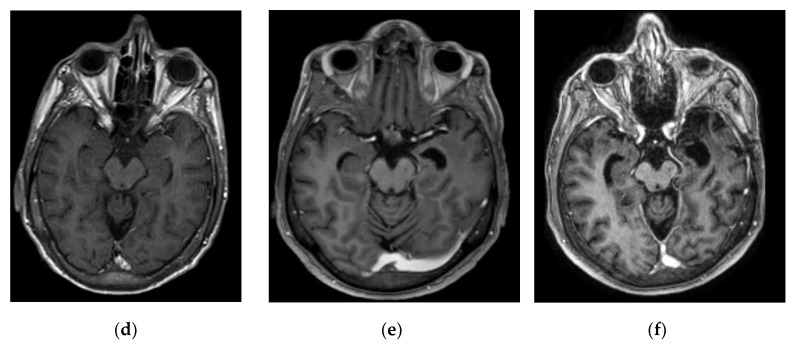
Neuroimaging: (**a**) FLAIR hyperintensity and swelling in bilateral hippocampi and temporal lobes; (**b**,**c**) Progressive worsening of FLAIR hyperintensities with associated atrophy at second and third admissions, respectively; (**d**) T1 hypointensities in bilateral hippocampi with subtle contrast enhancement; (**e**,**f**) Progressive worsening of T1 hypointensities without any contrast enhancement, with associated atrophy at second and third admissions, respectively.

Patient remained confused and agitated, but his metal status began to improve around the fifth day of admission, so dexmedetomidine was discontinued. He was transferred to general medicine floor. MRI brain with and without contrast obtained at this time showed T2/FLAIR hyperintensities in bilateral hippocampal and parahippocampal gyri, uncus, and temporal poles, with subtle patch contrast enhancement, more pronounced on the left without any restricted diffusion (Figure 1). CSF encephalitis panel was positive for herpes simplex virus type 2. Patient’s clinical presentation was consistent with herpes encephalitis. His acyclovir was continued, and other antibiotics were discontinued. Other CSF studies and infectious evaluations, including tick-borne illnesses, was negative. Patient continued to improve over the course of the admission, and he was discharged on the eleventh day of admission with IV acyclovir for a total of three weeks.

### 1.2. Admission 2

Six weeks later, patient presented with episodes of confusion, worsening gait, and insomnia for several days. Patient reportedly had improved after the previous discharge to the point that he was able to ambulate with a walker. He had completed three weeks of acyclovir and was taking his antiretroviral therapy (ART). About ten days prior to this presentation he started having gait issues and insomnia. Two days prior to this admission he started becoming more confused with brief episodes of unresponsiveness. On exam his vitals were normal, and he was awake but confused and agitated, occasionally combative, uttering profanities, and not following any commands. He was able to move all extremities spontaneously and localized pain. At this time a repeat MRI with and without contrast was obtained and showed worsening T2/FLAIR hyperintensities in bilateral temporal lobes compared to the prior exam (Figure 1). EEG only showed mild, generalized slowing and no seizures. Long-term video-EEG (VEEG) could not be obtained because patient was agitated and kept removing the leads. Patient’s levetiracetam was changed to valproic acid in light of agitation. A lumbar puncture was performed under anesthesia (given patient’s agitation and combativeness) and again showed lymphocytic pleocytosis: 16/mm^3^ (97% lymphocytes) without elevated glucose or protein. Patient was started on IV methylprednisolone for concerns of post-HSV autoimmune encephalitis, and acyclovir for concerns of incompletely treated HSV encephalitis. Patient was not given Intravenous Immunoglobulins (IVIG) because of liver cirrhosis, portal hypertension and acute kidney injury. Family declined plasmapheresis because he would have needed sedation and intubation for catheter placement and repeated cycles of plasmapheresis due to the severe agitation and combativeness. Extensive testing, including serum and CSF infection panels, was sent with all results negative. Serum and CSF autoimmune encephalitis panels were also sent and later resulted as negative. Patient was also started on antipsychotics per psychiatry’s recommendation. He was treated with IV acyclovir for three more weeks and had clinical improvement, but he did not return to his normal self. He continued to have aphasia, but he was not agitated. He was discharged home at the request of family who did not want to pursue further treatment. It was recommended that he return immediately if his condition worsened.

### 1.3. Admission 3

Four weeks later, he presented to an outside hospital with worsening mentation, hypoxemic respiratory failure, sepsis, and hematemesis. He reportedly had worsening dyspnea three days prior to presentation and subsequently developed fever and unresponsiveness. He was febrile (T 102.3 F), tachycardic (HR 128/min), tachypneic (RR 30/min), hypoxemic (SpO2 89%), and unresponsive, so he was intubated. Labs were significant for lactic acidosis of 4.6 mMol/L, hyperammonemia of 75 uMol/L, elevated procalcitonin of 4.78 ng/mL, hyponatremia of 132 mg/dL, creatinine of 1.2 mg/dL, AST of 168 IU/L, PT/INR of 15.1/1.5, and an ABG of 7.51/36.6/56/29.5. Urinalysis showed proteinuria, ketonuria, and leucocyte esterase. Chest radiograph showed confluent opacities in bilateral lower lung fields. CT head without contrast showed multiple areas of hypodensity in bilateral temporal lobes with diffuse mucosal thickening of all sinuses. He was then transferred to our hospital for further management.

**Figure 2:**
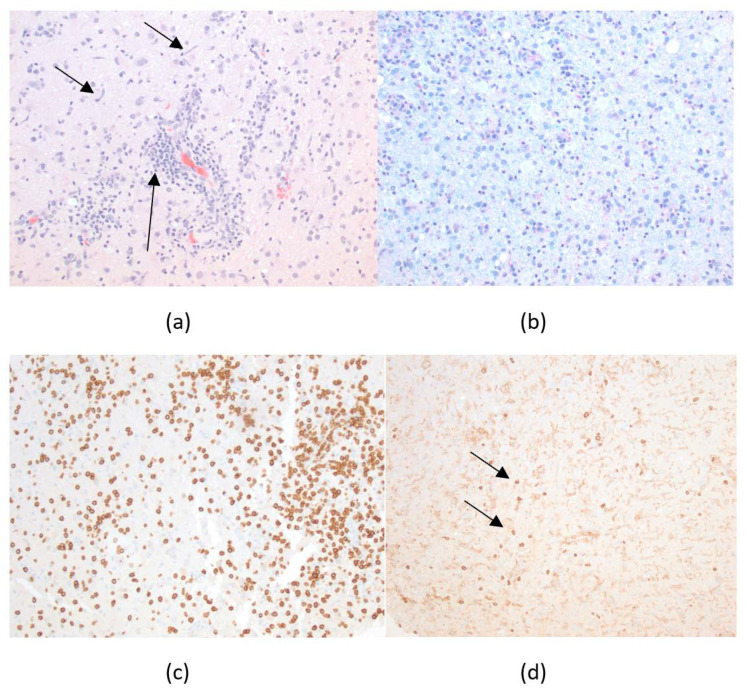
Histopathology: (**a**) An infiltrate of small, round lymphocytes is seen mainly around blood vessels (long arrow), and also scattered within the parenchyma on H&E stain. Prominent microglial proliferation (short arrows) is also present; (**b**) Essentially complete loss of myelin with no blue staining is seen on LFB/PAS stain; (**c**) Lymphocytic population is overwhelmingly comprised of CD8-positive T-cells on immunohistochemical staining; (**d**) CD4 highlights only an occasional CD4-positive T-cell (arrows) with a faint staining of microglial population in the background immunohistochemical staining.

A repeat lumbar puncture was performed and again showed lymphocytic pleocytosis: 21/mm^3^ (97% lymphocytes) with normal glucose and protein levels. CSF samples were again sent for infectious meningitis and encephalitis panels as well as autoimmune encephalitis testing. He was started on vancomycin and cefepime for pneumonia as well as lactulose for hyperammonemia. He developed thrombocytopenia (70 K) and was empirically given doxycycline for possible ehrlichiosis due to thrombocytopenia, elevated liver enzymes, hyponatremia, and the history of a tick bite. His sputum culture grew *Klebsiella pneumoniae*, which was sensitive to ceftriaxone. Consequently, patient was transitioned to ceftriaxone and other antibiotics were discontinued. Doxycycline was continued for possible ehrlichiosis. Patient continued to be on sedation because he became agitated and combative while weaning sedation. VEEG showed non-convulsive status epilepticus arising from the right fronto-temporal region. Patient was loaded with IV lacosamide and continued on valproic acid and maintenance therapy. His seizures resolved, and VEEG was discontinued after three days of monitoring. MRI brain with and without contrast revealed redemonstration of FLAIR hyperintensities involving bilateral hippocampal and parahippocampal gyri, insular and peri-insular regions, and bilateral frontal subcortical white matter without any interval worsening or post-contrast enhancement (Figure 1). Patient’s respiratory status improved, so he was extubated on the tenth day and transferred to the general medicine floor. He continued to be agitated and combative requiring restraints and was continued on scheduled quetiapine and lorazepam, as needed for agitation. Due to his continued encephalopathy and concern for autoimmune encephalitis, steroids and plasmapheresis were discussed with the family but they declined these therapies without a formal diagnosis. After extensive discussion with the family, a decision was made to obtain a brain biopsy. A stereotactic brain biopsy was obtained from the left temporal lobe. Pathology showed a polymorphous infiltrate of lymphocytes, and immunohistochemical staining revealed an overwhelming predominance of CD8+ T-cells in a largely perivascular distribution (Figure 2), consistent with CD8 encephalitis. Patient continued to be agitated and in restraints. Steroids were offered for treatment of the CD8 encephalitis, but it was declined by family due to concerns of exacerbating infections, lack of evidence of efficacy in this disease and exacerbating agitation. Patient was ultimately taken home by the family. He did not return to his follow-up appointments in the infectious disease or neurology clinics.

He had a telemedicine appointment with his primary care physician nine months later and had improved significantly by the time of this visit. He was calm and was able to form complete and meaningful sentences. He was able to perform activities of daily living with assistance.

## 2. Discussion

HIV is associated with a multitude of opportunistic infections of the CNS such as cryptococcus, tuberculosis, toxoplasma, CMV, EBV, JC virus etc., the majority of which are associated with a low CD4 count—typically less than 250 CD4 cells/μL [1,2,3,4]. They are less common in patients with a CD4 count greater than 500 cells/μL [5]. With the advent of highly effective ART, the overall incidence of HIV related opportunistic infections has decreased markedly over the last few decades [4,6]. Although mucocutaneous lesions with HSV are very common in patients with AIDS [4,5], HSV encephalitis in AIDS is not very common and both are usually seen in patients with a low CD4 count [5,7]. Our patient had well-controlled HIV but still developed HSV encephalitis. His first presentation was typical for viral encephalitis and was treated early antiviral therapy. Untreated HSV encephalitis has a mortality rate as high as 70–90% [8]. Hence, early diagnosis and prompt treatment of encephalitis is important to prevent long-term sequelae for the patient and to preserve life [4,9,10]. He had completed the course of antiviral therapy and clinically improved during the course of hospital admission.

He subsequently presented with worsening encephalopathy, seizures, and psychosis. At this presentation, diagnosis was not as straightforward and included incompletely treated HSV encephalitis, HIV encephalopathy, and NMDA related autoimmune encephalitis in the differential. NMDA encephalitis can occur as a complication following HSV encephalitis in about a third of patients [11]. This relationship has been established in human studies and animal models [12,13]. NMDA encephalitis and HSV encephalitis can appear similar on neuroimaging with involvement of the temporal lobes [14,15,16]. Given the confluent white matter disease, a diagnosis of HIV encephalopathy and HIV-related primary CNS vasculitis, which can manifest as acute to subacute encephalopathy with similar findings on neuroimaging, were also considered [17,18]. Progressive multifocal leukoencephalopathy (PML) and HIV related immune reconstitution syndromes can present with similar radiographic findings [19,20,21,22] but were unlikely in a patient with CD4 count of greater than 500/μL. In the clinical context of rapidly worsening encephalopathy and seizures, NMDA encephalitis and HSV encephalitis recrudescence was deemed more likely, and the patient was subsequently treated with antivirals and steroids, but family declined plasmapheresis at the time. In retrospect, the second admission was likely secondary to CD8 encephalitis, as proven by biopsy later during the third admission, and was likely caused by an immune response triggered by HSV encephalitis and subsequently improved with steroids given for presumed autoimmune encephalitis and worsened a few weeks after discontinuation of steroids.

CD8 encephalitis is a rare inflammatory disorder seen in HIV patients who are typically well-controlled with ART. It is characterized by infiltration of CD8+ T-cells into the brain parenchyma causing marked inflammation. It may manifest clinically with encephalopathy, seizures, coma, or even death [23,24]. Neuroimaging typically shows confluent T2/FLAIR hyperintensities in cortical and subcortical white matter [23,24,25]. Its pathogenesis is still unknown, but several factors such as CNS infections, discontinuation of ART, immune reconstitution inflammatory syndrome (IRIS), etc. in patients with HIV can trigger an immune response leading to CD8 encephalitis. It is a pathologically distinct process from HIV encephalitis and diffuse infiltrative lymphocytosis syndrome (DILS), which occur due to an immune response to high HIV viral load in CNS/PNS causing active inflammation [23,24]. It is hypothesized that in HIV-related illnesses there is an imbalance between CD8+ and CD4+ T-cells, which in the presence of certain triggers can lead to this inflammatory response with a predominance of CD8+ T-cells in the brain [23,26].

The clinical scenario was fairly challenging for our patient as HSV encephalitis and NMDA encephalitis, which are more common as compared to CD8 encephalitis and have similar presentation, were higher on differential diagnosis. Although other differential diagnoses as mentioned above were also considered, CD8 encephalitis was not amongst them. A brain biopsy was obtained during the third admission, which revealed the diagnosis of CD8 encephalitis and correlated with the imaging and clinical course. This highlights the challenge in making this diagnosis due to paucity of literature, with about 50 cases reported thus far, and is typically made on pathological specimen [23,25,26,27,28,29,30,31,32,33]. The natural history of this disease has not been well established, and a majority of the cases reported are by post-mortem pathology diagnosis [23]. Although there are no established treatments for CD8 encephalitis, steroids have been suggested to be useful in some cases, but the results have been variable [23,25,28,33]. Given this information, family declined further treatment given the lack of evidence and took the patient home. Patient, however improved to the point of being able to communicate in small sentences and being able to carry out some tasks of daily living without any further treatment other than ART. This points to the heterogeneity in presentation and outcome of the disease.

## 3. Conclusions

CD8 encephalitis is a rare cause of encephalitis in HIV patients and often is difficult to diagnose. It can be triggered by other CNS infections and persistent or fluctuating encephalopathy should prompt consideration of this disease in differential. There is no consensus on long-term management, but studies have suggested response to steroids in some patients.

## References

[1] Bowen L.N., Smith B., Reich D., Quezado M., Nath A. (2016). HIV-associated opportunistic CNS infections: Pathophysiology, diagnosis and treatment. Nat. Rev. Neurol..

[2] Tan I.L., Smith B.R., von Geldern G., Mateen F.J., McArthur J.C. (2012). HIV-associated opportunistic infections of the CNS. Lancet Neurol..

[3] Le L.T., Spudich S.S. (2016). HIV-Associated Neurologic Disorders and Central Nervous System Opportunistic Infections in HIV. Semin. Neurol..

[4] U.S. Department of Health and Human Services (2015). Guidelines for the Prevention and Treatment of Opportunistic Infections in HIV-Infected Adults and Adolescents: Recommendations from the Centers for Disease Control and Prevention, the National Institutes of Health, and the HIV Medicine Association of the Infectious Diseases Society of America.

[5] Panel on Guidelines for the Prevention and Treatment of Opportunistic Infections in Adults and Adolescents wih HIV (2021). Guidelines for the Prevention and Treatment of Opportunistic Infections in Adults and Adolescents with HIV.

[6] Matinella A., Lanzafame M., Bonometti M.A., Gajofatto A., Concia E., Vento S., Monaco S., Ferrari S. (2015). Neurological complications of HIV infection in pre-HAART and HAART era: A retrospective study. J. Neurol..

[7] Moulignier A., Baudrimont M., Martin-Negrier M.-L., Mikol J., Lapresle C., Dupont B. (1996). Fatal brain stem encephalitis due to herpes simplex virus type 1 in AIDS. J. Neurol..

[8] Bradshaw M.J., Venkatesan A. (2016). Herpes Simplex Virus-1 Encephalitis in Adults: Pathophysiology, Diagnosis, and Management. Neurotherapeutics.

[9] Ellul M., Solomon T. (2018). Acute encephalitis—Diagnosis and management. Clin. Med..

[10] Walensky R.P., Paltiel A.D., Losina E., Mercincavage L.M., Schackman B.R., Sax P.E., Weinstein M.C., Freedberg K.A. (2006). The survival benefits of AIDS treatment in the United States. J. Infect. Dis..

[11] Nosadini M., Mohammad S.S., Corazza F., Ruga E.M., Kothur K., Perilongo G., Frigo A.C., Toldo I., Dale R.C., Sartori S. (2017). Herpes simplex virus-induced anti-N-methyl-d-aspartate receptor encephalitis: A systematic literature review with analysis of 43 cases. Dev. Med. Child Neurol..

[12] Linnoila J., Pulli B., Armangué T., Planagumà J., Narsimhan R., Schob S., Zeller M.W.G., Dalmau J., Chen J. (2019). Mouse model of anti-NMDA receptor post–herpes simplex encephalitis. Neurol.-Neuroimmunol. Neuroinflamm..

[13] Morris N.A., Kaplan T.B., Linnoila J., Cho T. (2016). HSV encephalitis-induced anti-NMDAR encephalitis in a 67-year-old woman: Report of a case and review of the literature. J. Neurovirol..

[14] Bacchi S., Franke K., Wewegama D., Needham E., Patel S., Menon D. (2018). Magnetic resonance imaging and positron emission tomography in anti-NMDA receptor encephalitis: A systematic review. J. Clin. Neurosci..

[15] Heine J., Prüss H., Bartsch T., Ploner C., Paul F., Finke C. (2015). Imaging of autoimmune encephalitis–Relevance for clinical practice and hippocampal function. Neuroscience.

[16] Kastrup O., Wanke I., Maschke M. (2008). Neuroimaging of infections of the central nervous system. Semin. Neurol..

[17] Post M., Tate L.G., Quencer R.M., Hensley G.T., Berger J.R., Sheremata W.A., Maul G. (1988). CT, MR, and pathology in HIV encephalitis and meningitis. Am. J. Roentgenol..

[18] Gutierrez J., Ortiz G. (2011). HIV/AIDS patients with HIV vasculopathy and VZV vasculitis. Clin. Neuroradiol..

[19] Tan K., Roda R., Ostrow L., McArthur J., Nath A. (2009). PML-IRIS in patients with HIV infection: Clinical manifestations and treatment with steroids. Neurology.

[20] Smith A.B., Smirniotopoulos J.G., Rushing E.J. (2008). From the archives of the AFIP: Central nervous system infections associated with human immunodeficiency virus infection: Radiologic-pathologic correlation. Radiographics.

[21] Bag A.K., Curé J.K., Chapman P.R., Roberson G.H., Shah R. (2010). JC virus infection of the brain. AJNR Am. J. Neuroradiol..

[22] Johnson T., Nath A. (2011). Immune reconstitution inflammatory syndrome and the central nervous system. Curr. Opin. Neurol..

[23] Lucas S.B., Wong K.T., Nightingale S., Miller R.F. (2021). HIV-associated CD8 encephalitis: A UK case series and review of histopathologically confirmed cases. Front. Neurol..

[24] Lescure F.-X., Moulignier A., Savatovsky J., Amiel C., Carcelain G., Molina J.-M., Gallien S., Pacanovski J., Pialoux G., Adle-Biassette H. (2013). CD8 encephalitis in HIV-infected patients receiving cART: A treatable entity. Clin. Infect. Dis..

[25] Cheema A., Mathias K., Bui C., Dunham S.R., Goodman J.C., El Sahly H.M. (2019). CD8 Encephalitis in a Treatment-Naive and a Virologically Suppressed Patient with HIV. Can. J. Neurol. Sci./J. Can. Sci. Neurol..

[26] Miller R.F., Isaacson P.G., Hall-Craggs M., Lucas S., Gray F., Scaravilli F., An S.F. (2004). Cerebral CD8+ lymphocytosis in HIV-1 infected patients with immune restoration induced by HAART. Acta Neuropathol..

[27] Zarkali A., Gorgoraptis N., Miller R., John L., Merve A., Thust S., Jager R., Kullman D., Swayne O. (2017). CD8+ encephalitis: A severe but treatable HIV-related acute encephalopathy. Pract. Neurol..

[28] Kerr C., Adle-Biassette H., Moloney P., Hutchinson S., Cryan J.B., Clarke S., Mulcahy F., Devitt E. (2020). CD8 encephalitis with CSF EBV viraemia and HIV drug resistance, a case series. Brain Behav. Immun.-Health.

[29] Wood A.C., Parker R., Allinson K., Scoffings D. (2022). CD8 encephalitis presenting as autoimmune encephalitis in HIV-1 infection. BMJ Case Rep. C.P..

[30] Cheema A., Mathias K., Bui C., Dunham S.R., Goodman J.C., El Sahly H.M. (2020). CD8 Encephalitis in a Treatment-Naive and a Virologically Suppressed Patient with HIV–ERRATUM. Can. J. Neurol. Sci..

[31] Gray F., Lescure F.X., Adle-Biassette H., Polivka M., Gallien S., Pialoux G., Moulignier A. (2013). Encephalitis with infiltration by CD8+ lymphocytes in HIV patients receiving combination antiretroviral treatment. Brain Pathol..

[32] Ishiguro M., Ueno Y., Ishiguro Y., Takanashi M., Murai K., Taieb G., Daida K., Suda A., Yokoyama K., Naito T. (2020). CD8+ T-cell encephalitis mimicking PRES in AIDS: A case report. BMC Neurol..

[33] Salam S., Mihalova T., Ustianowski A., McKee D., Siripurapu R. (2016). Relapsing CD8+ encephalitis—Looking for a solution. Case Rep..

